# Patient and doctor perspectives on HIV screening in the emergency department: A prospective cross-sectional study

**DOI:** 10.1371/journal.pone.0180389

**Published:** 2017-07-21

**Authors:** Noemy De Rossi, Nicolas Dattner, Matthias Cavassini, Solange Peters, Olivier Hugli, Katharine E. A. Darling

**Affiliations:** 1 Faculty of Biology and Medicine, University of Lausanne, Lausanne, Switzerland; 2 Infectious Diseases Service, Lausanne University Hospital, Lausanne, Switzerland; 3 Department of Oncology, Lausanne University Hospital, Lausanne, Switzerland; 4 Emergency Department, Lausanne University Hospital, Lausanne, Switzerland; Waseda University, JAPAN

## Abstract

**Background:**

The emergency department (ED) is mentioned specifically in the Swiss HIV testing recommendations as a site at which patients can benefit from expanded HIV testing to optimise early HIV diagnosis. At our centre, where local HIV seroprevalence is 0.2–0.4%, 1% of all patients presenting to the ED are tested for HIV. Barriers to HIV testing, from the patient and doctor perspective, and patient acceptability of rapid HIV testing were examined in this study.

**Methods:**

Between October 2014 and May 2015, 100 discrete patient-doctor encounter pairs undertook a survey in the ED of Lausanne University Hospital, Switzerland. Patients completed a questionnaire on HIV risk factors and were offered free rapid HIV testing (INSTI^™^). For every patient included, the treating doctor was asked if HIV testing had 1) been indicated according to the national testing recommendations, 2) mentioned, and 3) offered during the consultation.

**Results:**

Of 100 patients, 30 had indications for HIV testing through risk factors or a suggestive presenting complaint (PC). Fifty patients accepted rapid testing; no test was reactive. Of 50 patients declining testing, 82% considered themselves not at risk or had recently tested negative and 16% wished to focus on their PC. ED doctors identified 20 patients with testing indications, mentioned testing to nine and offered testing to six. The main reason for doctors not mentioning or not offering testing was the wish to focus on the PC.

**Discussion:**

Patients and doctors at our ED share the testing barrier of wishing to focus on the PC. Rapid HIV testing offered in parallel to the patient-doctor consultation increased the testing rate from 6% (offered by doctors) to 50%. Introducing this service would enable testing of patients not offered tests by their doctors and reduce missed opportunities for early HIV diagnosis.

## Background

Early diagnosis and treatment of HIV infection optimises the benefit of antiretroviral therapy and prevents onward transmission. In Switzerland, while 50.2% of patients presenting with HIV infection are diagnosed promptly, the remainder are diagnosed late, with CD4 counts below 350 cells/mm^3^ or an AIDS-defining event [1, 2].

HIV testing strategies can be characterised as 1) *non-targeted*, whereby individuals seeking healthcare are offered an HIV test regardless of their symptoms and signs and regardless of their risk-factor profile for HIV acquisition, or 2) *targeted*, whereby the offer of HIV testing is restricted to individuals presenting symptoms and signs suggestive of HIV-associated indicator conditions (ICs), those with conditions in whom HIV infection should be excluded, such as pregnancy or prior to commencing immunosuppressant therapy, or those considered at risk of HIV acquisition. *Diagnostic* testing refers to the specific situation in which testing is performed to confirm rather than exclude HIV infection and can be considered as a sub-type of targeted testing. In addition, patient consent to testing can be *opt-out*, where testing is performed unless the patient explicitly declines, or *opt-in*, where testing is offered and then performed if the patient accepts.

In Switzerland, the Federal Office of Public Health (FOPH) recommends targeted and diagnostic HIV testing and highlights the emergency department (ED) as a service in which testing should occur [3]. The FOPH recommendations, first published in 2007 and updated in 2010, 2013 and 2015 [3–6], comprehensively list five main groups of HIV testing indications: symptoms and signs of acute HIV infection; conditions pathognomonic of AIDS; conditions in which HIV infection occurs in >0.5% of cases (ICs); conditions in which HIV infection should be excluded; and populations in whom the risk of HIV acquisition is high (men who have sex with men (MSM), injecting drug users (IDUs), individuals from countries with high HIV seroprevalence, notably sub-Saharan Africa, and those with sexual partners from a high-risk group). The recommendations refer to physician-initiated counselling and testing (PICT) in which the health care provider must initiate the offer of HIV testing when it is indicated; consent to testing is opt-in.

Despite the FOPH recommendations, the HIV testing rate in the ED at our centre is around 1% of all patients seen [7]. Indeed, even in EDs in the United States (US), where the Centers for Disease Control and Prevention (CDC) recommend non-targeted, opt-out testing [8], physician-initiated testing has resulted in low (<1%) HIV testing rates [9]. Several barriers to HIV testing in the US ED setting have been reported. *Patient* barriers include the belief that testing is unnecessary, through lack of risk or a recent negative test [10], the wish to focus on the reason for presenting to the ED [10], and concerns regarding confidentiality [10, 11], while *doctor* barriers involve competing priorities [12], confidentiality [11], time [13, 14] and space [13], concerns regarding follow-up care [13, 14], and the perception that HIV testing is not part of emergency care provision [13]. Many of these studies were published in settings where the main method of HIV testing was a laboratory-based test. More recently, the implementation of rapid HIV testing on oral fluid samples has been reported to increase testing rates [15]. In the United Kingdom (UK), HIV testing on oral fluid samples was successfully implemented in settings in which the UK national HIV testing guidelines recommend non-targeted testing (where local diagnosed HIV prevalence is greater than 2 /1000 persons) by providing supplementary personal to initiate testing [16]. Another means of increasing HIV testing rates in the ED is to add-on HIV testing to existing blood draws [17].

In Switzerland, the health care system, patient population and HIV testing recommendations differ from those of the US and the UK, and HIV testing barriers may also differ. The primary aim of this study was to examine patient and doctor barriers to HIV testing in the ED, in a setting in which universal (non-targeted) testing is not recommended. Two secondary aims were to examine 1) doctor capacity to identify patients with HIV testing indications and 2) patient acceptance of rapid HIV testing.

## Methods

### Ethics statement

The study was approved by the Ethical Committee on Human Research of the Canton of Vaud, Switzerland (protocol number 95/14). Participants gave written consent prior to both study inclusion and rapid HIV testing.

### Study setting and participants

The study took place between 1 October 2014 and 19 May 2015 in the ED of Lausanne University Hospital (LUH), Lausanne, Switzerland. LUH ED provides around 40,000 consultations per year [18] and HIV seroprevalence in the local population is 0.2–0.4% [19, 20]. In this setting, HIV testing is performed on venous blood using a laboratory-based fourth generation screening assay which identifies anti-HIV-1/2 antibodies (IgG and IgM) and the p24 antigen (Cobas Elecsys HIV combi PT, Roche Diagnostics, Switzerland). Reactive samples undergo a neutralisation assay for the p24 antigen (Cobas Elecsys HIV Ag Confirmatory Test, Roche Diagnostics), a line immunoassay (INNO-LIA^™^ HIV I/II Score, Innogenetics NV) and a plasma viral load determination (Cobas AmpliPrep/Cobas TaqMan HIV-1, version 2.0, Roche Diagnostics) before they are released as positive for HIV. A result is available in 120 minutes (during working hours) or the following day. At the time of this study, rapid HIV testing was not part of standard practice at LUH ED.

The study participants were 1) ED patients aged 18–75 years old and 2) ED doctors treating included patients. Exclusion criteria (patients) were clinical instability, transfer from another hospital / prison, admission >12 hours prior to inclusion, known positive HIV status and inability to provide informed consent. Doctors had no exclusion criteria but participation was restricted to a maximum of four questionnaires per shift to avoid behaviour change (Hawthorne effect).

### Study design

The study was prospective and cross-sectional with a convenience sample of 100 discrete patient-doctor pairs who undertook a face-to-face survey using a paper questionnaire (S1 Text. Questionnaire). Patient inclusion, patient and doctor surveys, questionnaire response documentation and rapid testing of patients were performed by two final-year medical students (NDR and ND, referred to as the *study investigators*) as part of a Masters thesis. Such a thesis requires 400 hours of work and for this reason the convenience sample was limited to 100 patient-doctor pairs to ensure time to conduct the study, analyse the results and produce a manuscript whilst continuing to fulfil their medical study commitments.

**Fig 1:**
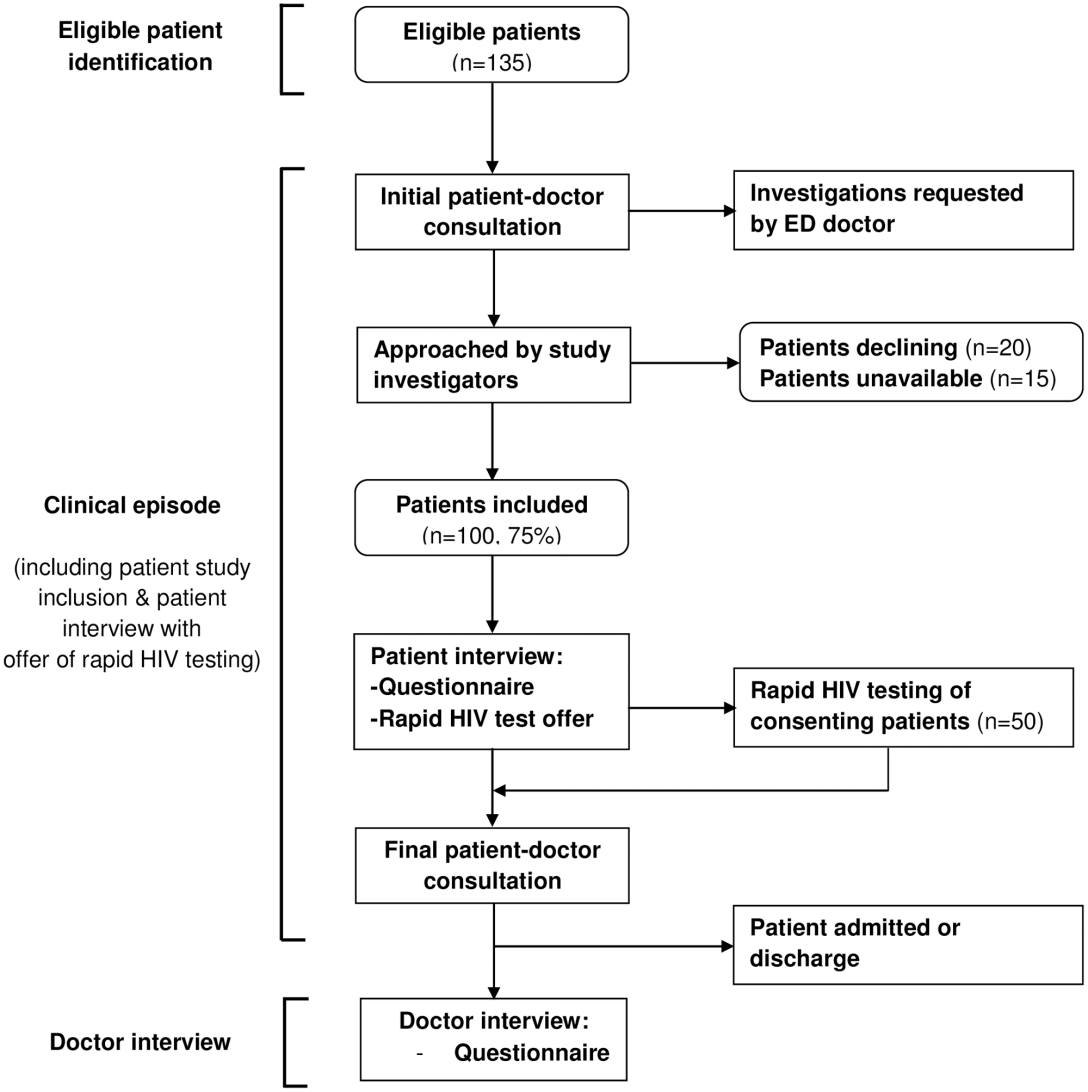
Flow chart showing the sequence of patient inclusion, study patient participation (questionnaire and rapid HIV testing) and doctor participation (questionnaire). Boxes with rounded corners indicate numbers; those with squared corners indicate processes.

The study investigators identified eligible patients using the live ED computer system and approached patients after their initial patient-doctor consultation, having confirmed with the relevant ED doctor that this was convenient (Fig 1).

The study investigators conducted the study over 36 shifts. During a single shift, they surveyed between zero and eight patient-doctor pairs, depending on patient eligibility and availability. Patients consenting to participate completed a questionnaire (see below) and were offered a free rapid HIV test. The ED doctor of each included patient completed the doctor questionnaire within 24 hours of the clinical episode, the period from the initial to the final patient-doctor consultation (Fig 1). Prior to the study, ED doctors attended training seminars covering the latest FOPH testing recommendations published at the time of the study [21], the practice of testing and the study protocol, and were given a pocket-sized card summarising the recommendations (S2 Text. Recommendations).

The patient questionnaire had three sections and took approximately 15 minutes to complete. Section 1 covered demographic details and the reason for presentation (presenting complaint, PC), HIV testing history, and HIV risk factors derived from the FOPH testing recommendations [21]. In Section 2, patients were asked if they would have liked to have been offered HIV testing and, where applicable, the reasons they did not mention HIV themselves. In Section 3, patients were offered rapid HIV testing using fingertip blood (24 INSTI^™^ HIV-1/HIV-2 Rapid Antibody Test, BioLytical Laboratories, Richmond, BC, Canada) and invited to give reasons for accepting or declining testing from a list of response options. Patient surveys occurred between 08:00H and 16:00H to ensure access to a duty infectious diseases physician in the event of a reactive test.

The doctor questionnaire covered demographic details and postgraduate experience then asked doctors 1) if HIV testing had been indicated according to the FOPH testing recommendations, 2) if they had mentioned HIV and 3) if they had offered testing, providing reasons in each case from a list of options.

The questionnaire formats were analogous to those of questionnaires previously employed locally [22, 23] and nationally (www.lovelife.ch, a public information site). A pilot study was conducted among patient-doctor pairs at LUH ED throughout October 2014 to validate the questionnaires for content and to confirm practical feasibility of rapid testing.

### Data management and statistical analysis

Data from the paper questionnaires were entered independently into two separate databases by each of the two investigators. The databases were then merged and discrepancies were resolved by checking the original questionnaire to ensure data accuracy [24].

For analysis, patients were grouped by HIV risk according to FOPH testing recommendations as described in the Background. Group A patients gave a PC suggestive of primary HIV infection; group B patients presented HIV risk factors and/or condomless sex with sexual partner(s) with risk factors; group C patients reported condomless sex during the preceding six months but no other risk factors; group D patients reported no risk factors. Rates of HIV testing offered, accepted and performed were calculated for ED doctors and the study investigators.

Data are presented as mean ± standard deviation (SD), median and inter-quartile range (IQR) and as percentages. Proportions were compared using the Chi-squared test, or Fisher’s exact test when appropriate. Statistical analyses were conducted using Microsoft Excel 2010 (Microsoft Corporation, Redmond, WA, USA).

## Results

### Patients

Between 11 November 2014 and 19 May 2015, 135 eligible patients were identified of whom 100 (75%) were available to participate (Fig 1). The main obstacle to including patients was not being able to confirm with the relevant ED doctor that this was convenient. Of patients ineligible (at least one patient per shift), the main reason was age being over 75 years, followed by inability to provide informed consent (acute psychiatric episode, intoxication and/or inability to speak French) and clinical instability.

As 100 patient-doctor pairs were included, participant numbers and percentages are presented interchangeably unless subgroups are described (Table 1).

**Table 1:** Patient characteristics, in total and by HIV risk group.

	All patients (n = 100)	Group A/B (n = 30)	Group C (n = 48)	Group D (n = 22)	*P* value^5^
**Age (years), mean (SD)**	39.9 (13)	37.7 (12)	40.8 (13)	41 (15)	0.9
**Nationality, n (%)**					
Switzerland	56 (56)	11 (37)	27 (56)	18 (82)	0.17
Europe	28 (28)	10 (33)	17 (35)	1 (4.6)	
Other	16 (16)	9 (30)	4 (8.3)	3 (14)	
**Male sex, n (%)**	65 (65)	18 (60)	34 (71)	13 (60)	0.9
**Low-acuity patients**^1^**, n (%)**	88 (88)	24 (80)	44 (92)	20 (91)	0.31
**Discharged, n (%)**	77 (77)	21 (70)	40 (83)	16 (73)	0.88
**Risk factors**^2^**, n (%)**					
MSM	2 (2)	2 (6.7)	-	-	NA
Bisexual	3 (3)	3 (10)	-	-	
IDUs (current or former)	2 (2)	2 (6.7)	-	-	
Sub-Saharan African origin	6 (6)	6 (20)	-	-	
CS with high risk partner	18 (18)	18 (60)	-	-	
**Followed by family doctor, n (%)**	80 (80)	25 (83)	38 (79)	17 (77)	0.9
**CS in past six months**^3^**, n (%)**					
Yes (≥1 sexual partner)	68 (68)	20 (67)	48 (100)	NA	-
With stable partner only	62 (91)	16 (80)	46 (96)		0.36
**HIV testing history**^4^**, n (%)**					
≥1 previous test	66 (66)	22 (73)	36 (75)	8 (36)	0.03
Tested within past 12 months	26 (39)	11 (50)	13 (36)	2 (25)	-
**Attitude to HIV testing at ED visit**					
Wishing to discuss HIV with doctor	17 (17)	6 (20)	8 (17)	3 (14)	0.9
Accepting rapid HIV testing	50 (50)	14 (47)	26 (54)	10 (46)	0.9

^1^Low-acuity patients had minor injuries or complaints not requiring regular monitoring of vital signs and/or neurological status.

^2^Risk factors were those defined in the Swiss Federal Office of Public Health HIV testing recommendations (5). As some patients had HIV risk factors themselves *and* reported condomless sex (CS) with sexual partners with risk factors, the total number of patients exceeds the number of patients in group A/B;

^3^Patients reporting CS solely with a stable partner are presented as a percentage of patients reporting CS;

^4^Patients tested within the past 12 months are presented as a percentage of those tested;

^5^Group A/B is used as the reference when comparing patient risk groups unless stated otherwise.

Abbreviations: SD, standard deviation; ED, emergency department; MSM, Men who have sex with men; IDUs, injecting drug users; CS, condomless sex.

Most patients (83%) had not considered HIV testing during their ED consultation and gave not being at risk and wishing to focus on their PC as the main reasons (Table 2).

**Table 2:** Patient reasons for not mentioning HIV during the consultation with their emergency department doctor.

Reasons for not mentioning HIV	Patients who did not wish to discuss HIV with the ED doctor, n (%) (n = 83)	Patients who wished to discuss HIV but did not bring up the subject, n (%) (n = 17)
Wish to focus on PC	51 (62)	10 (59)
Patient not at risk	72 (87)	8 (47)
Forgot	NA	6 (35)
Recent negative test	4 (4.8)	NA
Feel embarrassed or afraid	22 (27)	1 (5.9)
Do not want to bother the doctor	NA	1 (5.9)
Concerned about confidentiality	NA	0 (0)

As multiple responses were allowed, the total number for each column is greater than the number of participants in each group.

Abbreviations: ED, emergency department; PC, presenting complaint; NA, not applicable.

NA refers to the reason not being listed as an option in the specific questionnaire section.

The 17 patients who would have liked to have been tested gave the reasons of wishing to focus on the PC and not being at risk as the main reasons for not mentioning HIV to their ED doctor (Table 2).

Fifty patients accepted rapid testing offered by the study investigators, through wishing to confirm negative HIV status (84%), to benefit from free testing (10%), and concern of being at risk (6%) (single response allowed); no test was reactive. Patients declining rapid testing believed themselves not at risk (56%), had recently tested negative (26%), preferred to focus on the PC (16%) or feared needles (one patient, 2%) (single response allowed).

### Doctors

All ED doctors (total 33) treating the 100 included patients agreed to participate. Participating ED doctors were predominantly junior grade (93%) with median postgraduate experience three years (IQR 1:3.5). The median number of questionnaires completed by a single ED doctor was two (IQR 2:4; range 2–9), and the median number completed within a single shift was one (IQR 1:2; range 1–4). Questionnaires were completed by each doctor during the same shift in which the patient-doctor consultation had taken place in all but five cases (in which the questionnaire was completed within 24 hours of the consultation). Doctors identified FOPH testing indications in 20 patients, including two identified erroneously (‘vaginal candidiasis’ and ‘intuition’). *Sensitivity* for identifying patients with testing indications (groups A/B) was 30% and *specificity* was 87%.

**Fig 2:**
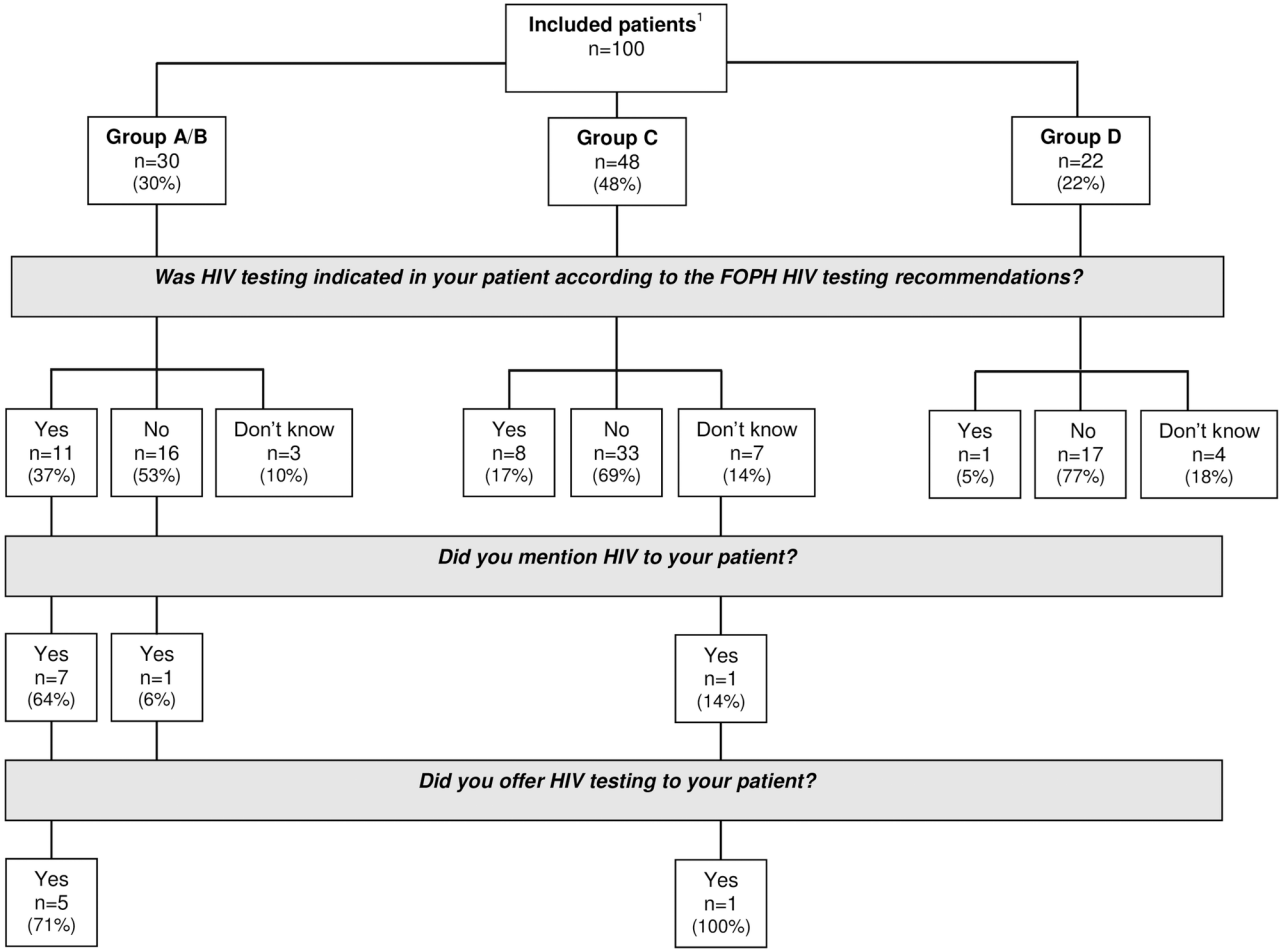
Flow chart showing the identification of Federal Office of Public Health (FOPH) indications for HIV testing, mention of HIV and offer of HIV testing by emergency department (ED) doctors, presented according to patient HIV risk group. ^1^Group A: patients with a reason for presenting suggestive of primary HIV infection; Group B: patients presenting HIV risk factors and/or reporting condomless sex with sexual partner(s) with risk factors; Group C: patients reporting condomless sex but no other risk factors; Group D: patients reporting no risk factors.

By the end of the clinical episode, doctors mentioned HIV to nine patients and offered testing to six (Fig 2).

The most common reason for not mentioning HIV or offering testing was wishing to focus / remain relevant to the PC (Table 3).

**Table 3:** Doctor reasons for not mentioning HIV and not offering testing to patients when indicated according to the Federal Office of Public Health (FOPH) testing recommendations.

Reasons for not mentioning HIV or not offering HIV testing	Doctors who did not mention HIV, n (%) (n = 91)	Doctors who did not offer testing, n(%) (n = 15)
Not relevant to PC	65 (71)	7 (47)
Wish to focus on PC	40 (44)	3 (20)
Patient not at risk	27 (30)	NA
HIV testing not indicated	24 (26)	NA
Have more urgent care to provide	20 (22)	0 (0)
Forgot	0 (0)	4 (27)
Proposed testing elsewhere	NA	4 (27)
Recent negative test	NA	3 (20)
Concerned about confidentiality	0 (0)	0 (0)

As multiple responses were allowed, the total number for each column is greater than the number of participants in each group.

Abbreviations: ED, emergency department; PC, presenting complaint; NA, not applicable.

NA refers to the reason not being listed as an option in the specific questionnaire section.

There was no difference in testing behaviour associated with the number of times a doctor participated or with particular time points during the study (data not shown). The patients offered HIV testing were seen by six different doctors.

Each study investigator performed rapid HIV testing on 50% of the patients they questioned. There was no difference in the demography of patients recruited by each investigator and no difference in HIV testing rates with time (*P*>0.9).

## Discussion

This is the first study exploring patient and doctor barriers to HIV testing in the Swiss ED setting. Both patients and doctors wished to focus on the PC over discussing HIV. Whilst medically valid, this shared wish was also a shared *barrier* to testing, as 75% of doctors who identified testing indications failed to offer testing. A further doctor barrier was the low (30%) sensitivity for identifying patients with testing indications, despite training seminars and pocket information cards. These findings are concerning if doctors are expected to initiate testing in line with FOPH recommendations.

Not being at risk was cited by both patients and doctors. The patients in this study were mainly Swiss or European. Importantly, there were no significant differences in age, sex, nationality (European or non-European) or access to primary health care between the different HIV risk groups. There are therefore no demographic markers arising from this patient sample which could be applied to the general ED population to enable easy identification of at-risk patients. To do this, it is necessary to take a detailed and specific sexual history, particularly as self-perceived, or self-reported, risk may not equate to true risk [1, 25, 26]. Indeed, among ED patients at one US urban centre, patients declining HIV testing had an almost three-fold higher rate of undiagnosed HIV infection [27]. As sexual history taking may be time-consuming, this step is often omitted by doctors seeing patients with unrelated complaints in settings with less time pressure than an ED [28, 29].

Rapid testing offered in parallel to the patient-doctor consultation circumvents the barrier of incompletely assessing HIV risk. Further, by initiating a dialogue, it is clear to the patient which test is being offered. In the current study, rapid testing was accepted by 50% of patients, including patients who did not wish initially to be tested. It is noteworthy that none of the patients who initially wished to be tested during the patient-doctor consultation mentioned HIV if the subject was not broached by the ED doctor. Conversely, the mention of HIV and offer of testing by the study investigators in parallel to the ED consultation resulted in 41% of the 83 patients who did not wish to be tested initially, agreeing to rapid HIV testing. These observations suggest that testing rates can be optimised by actively mentioning HIV and offering testing rather than waiting for the patient to ask. Another argument for an active dialogue is the previous observation at our centre that some patients believe erroneously that having a blood test means they have been tested for HIV, even if HIV was not mentioned by their doctor [23, 30]. As recent HIV testing was one of the main reasons for declining testing in the current study, it is possible that an erroneous belief of having been tested previously could lead to reticence towards ‘repeat’ testing and delayed HIV diagnosis in positive patients.

Although this study was not powered to compare HIV testing rates between doctors and the study investigators, the latter offering non-targeted testing as part of the study protocol and the former conducting targeted testing when this was considered to be indicated, the difference in testing rates occurring during the patient-doctor consultation and through rapid testing was marked: 6% versus 50%. Rather than informing ED doctors about HIV testing recommendations, testing could be offered by non-medical health care personnel working in parallel to the medical consultation. Indeed, the 2013 and 2015 FOPH recommendations state explicitly that the testing directive applies not only to doctors but to medical personnel in collaboration with doctors [5, 6]. A potentially more cost-effective approach would be to employ electronic devices, such as tablet computers [31]. These could be issued to patients as they wait to be seen, asking questions on risk factors for HIV acquisition, similar to the paper questionnaire in this study but without the need for additional personnel. In the context of HIV risk factor screening, the use of tablets has been reported to be well-accepted by patients, and may provide more accurate data on high risk behaviour than face-to-face surveys [32, 33].

This study has limitations. First, only clinically stable patients aged 18 to 75 years old were eligible, so most patients were recruited from the low-acuity section. Our findings therefore cannot be applied to the whole ED population, although older patients (over 75 years old) are more frequently clinically unstable [34]. In this way, offering HIV testing to low-acuity patients in parallel to the patient-doctor consultation does not replace routine, non-targeted testing and could potentially miss undiagnosed HIV in more unwell patients. Second, patients might have been grouped incorrectly by risk factor. Patients in group A had one or a combination of fever, flu-like symptoms or lymphadenopathy, because only the main PC was documented in the questionnaire, whereas the FOPH recommendations propose testing in patients with at least two of these symptoms. However, as only six patients belonged to group A, reclassification would have a marginal effect. Group B patients not engaging in high-risk behaviour since their last negative HIV test would have met criteria for grouping as C or D. However, whilst this might overestimate the number of patients ‘missed’ for testing, it does not alter the number identified as having testing indications but not offered testing. Third, needing to confirm that it was convenient for the ED doctor to conduct the patient survey slowed patient inclusion and, as the surveys were conducted during working hours, the patient sample studied may not be representative. However, the latter bias is limited as patients were eligible if admitted within 12 hours. Moreover, whilst selection bias might influence patient testing uptake, it would not influence doctor testing approach. Finally, although rapid testing uptake might increase through being free, only five patients gave cost as their main motivation.

In conclusion, patients and doctors share the barrier to HIV testing of wishing to focus on the PC. The offer of rapid HIV testing in parallel to the patient-doctor consultation was acceptable to patients, performed without complication and resulted in a testing rate of 50%. Offering non-targeted rapid HIV testing in our ED would enable testing of patients who may present HIV risk factors but would not otherwise be tested during their visit. An analysis of the cost-effectiveness of introducing such a measure using electronic tablets, against new HIV diagnoses likely to be made, is currently underway.

## Supporting information

S1 Text Recommendations. (DOC)

S2 Text Questionnaire. (DOC)

S1 Table Study database. (XLSX)

## Abbreviations

CS: condomless sex
ED: emergency department
FOPH: Federal Office of Public Health
IDUs: injecting drug users
MSM: Men who have sex with men
PC: presenting complaint
