# Supplementary material for: Patient and doctor perspectives on HIV screening in the emergency department: A prospective cross-sectional study
Source: PLoS One. 2017 Jul 21;12(7):e0180389. doi: 10.1371/journal.pone.0180389 (PMC5521743; doi:10.1371/journal.pone.0180389)
Supplement: S1 Text — (DOC) [file pone.0180389.s001.doc]

[
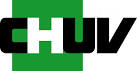
](http://www.google.ch/url?q=http://www.cliniquedudos.ch/fr/cv.html&sa=U&ei=BmAwU_z2Duiy7Ab2q4CoBg&ved=0CC8Q9QEwAQ&usg=AFQjCNHJiHnIRTgaIj_KqBXVvvkc3FrczA)
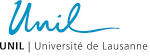


**Patient questionnaire**

| Label | Acceptation of rapid HIV testing in the emergency department | Number: | |
| --- | --- | --- | --- |
|  | Master: | Date:  _ _ /_ _ /_ _ | Time:  _ _ : _ _ |

**Patient data**

| 1.1. Hospital episode no: |  | | | |
| --- | --- | --- | --- | --- |
| 1.2. Date of birth: | _ _ /_ _ /_ _ _ _ | | | |
| 1.3. Nationality: | Switzerland (0) |  | South / Central America (3) |  |
|  | Europe (1) |  | North America (4) |  |
|  | Africa (2) |  | Asia (5) |  |
|  | Country: | | | |
| 1.4. Sex: | Male (0) |  | Female (1) |  |
| 1.5. Civil status: | Married (0) |  | Divorced (2) |  |
|  | Single (1) |  | Widower (3) |  |
| 1.6. Presenting complaint: | ETS code: |  |  |  |
| 1.7. ED section: | Medical majors (0) |  | Minors (1) |  |
|  | Surgical majors (2) |  |  |  |
| 1.8. Destination: | Discharge (0) |  | Admitted (1) |  |
| 1.9. Risk factors: | None (0) |  | Bisexual (3) |  |
|  | Heterosexual (1) |  | Injecting drug use (4) |  |
|  | Men who have sex with men (2) |  | Other (5) |  |

**Inclusion criteria**

| 2.1. Eligible : | Yes (1) |  | 2.2. | | | Accept (1) |  | Decline (0) | |  |
| --- | --- | --- | --- | --- | --- | --- | --- | --- | --- | --- |
|  |  |  |  |  |  |  |  |  |  |  |
|  | No (0) |  | 2.3 | Reason: | | Age < 18 years | | | (0) |  |
|  |  |  |  | | | Age > 75 years | | | (1) |  |
|  |  |  |  | |  | Not seen by a doctor | | | (2) |  |
|  |  |  |  | |  | Admitted > 12 hours | | | (3) |  |
|  |  |  |  | |  | Unstable clinical state[[1]](#footnote-2) | | | (4) |  |
|  |  |  |  | |  | Transferred from another hospital | | | (5) |  |
|  |  |  |  | |  | Cognitive disorders | | | (6) |  |
|  |  |  |  | |  | Mental retardation | | | (7) |  |
|  |  |  |  | |  | Acute alcohol intoxication | | | (8) |  |
|  |  |  |  | |  | Other substance intoxication[[2]](#footnote-3) | | | (9) |  |
|  |  |  |  | |  | Acute psychosis | | | (10) |  |
|  |  |  |  | |  | Suicide attempt | | | (11) |  |
|  |  |  |  | |  | Hearing impairment | | | (12) |  |
|  |  |  |  | |  | Non-francophone without interpreter | | | (13) |  |
|  |  |  |  | |  | HIV positive | | | (14) |  |
|  |  |  |  | |  | Prisoner | | | (15) |  |
|  |  |  |  | |  | Other | | | (16) |  |

|  | Acceptation of rapid HIV testing in the emergency department | Number: |
| --- | --- | --- |

Thank you for agreeing to participate in this study.

We are interested in the HIV testing approaches in the emergency department.

In this questionnaire, we wish to explore your opinion on HIV testing. If you do not wish to answer to one or several questions, you can move on to the following at any time.

| 1. *What brought you to the emergency department?*   *(presenting complaint)* |  | |
| --- | --- | --- |
| 1. *Do you have a family doctor?* | Yes (1)  No (0) |  |
|  |  |  |
| 1. *When did you last see him/her?* |  |  |
| 1. *Do you know your diagnosis today?* | Yes (1)  No (0) |  |
|  |  |  |
| 1. *Did the doctor or doctors you have seen talk about HIV or HIV screening?* | Yes (1)  No (0) |  |
|  |  |  |
| 1. *If he/they did, did he/they offer an HIV test?* | Yes (1)  No (0) |  |
|  |  |  |
| 1. *As part of your ED visit today, would you have liked to be tested for HIV?* | Yes (1)  No (0) |  |
|  |  |  |
| 1. *If yes, for what reasons?* | Unprotected sex (a) |  |
|  | I think I am at risk for HIV (b) |  |
|  | I want to be tested (c) |  |
|  | I want to make sure I am HIV negative (d) |  |
|  | Other (e):  …………………………………… |  |
| 1. *If not, for what reasons?* | I do not think I am at risk for HIV (a) |  |
|  | I don’t think it relevant for the reason I came to the ED (b) |  |
|  | I don’t have sex anymore (c) |  |
|  | I don’t want to be tested (d) |  |
|  | I’m afraid to be tested (e) |  |
|  | I’ve been recently tested (f) |  |
|  | Other (g):  …………………………………… |  |
| 1. *If you wanted to talk about HIV and your doctor didn’t mention the subject, why didn’t you bring the subject up?* | I prefer to talk about this to another doctor (a) |  |
|  | I prefer to focus on the reason I came to the ED (b) |  |
|  | I don’t want to bother the doctor with several issues at the same time (c) |  |
|  | I don’t want to talk about my private life (d) |  |
|  | I have concerns about confidentiality in the ED (e) |  |
|  | HIV doesn’t concern me (f): |  |
|  | I feels too embarrassed to bring up the subject (g) |  |
|  | I didn’t think to bring up the subject (h) |  |

Now a few questions about your HIV risk factors. We remind you that you can move on to the next question if you do not wish to answer to a particular question.

| - 1. *Have you ever been tested for HIV?* | Once(1) | | |  |
| --- | --- | --- | --- | --- |
|  | Several times (2) | | |  |
|  | Never (0) | | |  |
|  | Don’t want to answer (3) | | |  |
| - 1. *If at least once, when was the last time?* | During the last month (1) | | |  |
|  | During the last six months (2) | | |  |
|  | A year ago (3) | | |  |
|  | More than a year ago (4) | | |  |
|  | Don’t want to answer (5) | | |  |
| - 1. *If at least once, where were you tested last time?* | Family doctor (1) | | |  |
|  | Maternity (2) | | |  |
|  | Anonymous screening centre (3) | | |  |
|  | Medical outpatient clinic (4) | | |  |
|  | Emergency department (5) | | |  |
|  | Walk-in emergency centre (6) | | |  |
|  | Myself with a kit bought online (7) | | |  |
|  | Private clinic (8) | | |  |
|  | During an hospital stay (9) | | |  |
|  | Army (10) | | |  |
|  | Blood donation (11) | | |  |
|  | Private laboratory (12) | | |  |
|  | Urologist (13) | | |  |
|  | Occupational medicine (14) | | |  |
|  | Immunology (15) | | |  |
|  | Don’t remember (16) | | |  |
|  | Don’t want to answer (17) | | |  |
| - 1. *a) Have you had unprotected sex during the last six months?* | Once (1) | | |  |
|  | Several times (2) | | |  |
|  | Never (0) | | |  |
|  | Don’t want to answer (3) | | |  |
| - - *b) If yes, with what type of partner?* | Stable (0) | | |  |
|  | Occasional (1) | | |  |
|  | Both (2) | | |  |
|  | Don’t want to answer (3) | | |  |
| - - *c) If yes, do you know the HIV status of your partner(s)?* | Yes (1) | | |  |
|  | No (0) | | |  |
|  | Don’t want to answer (2) | | |  |
| - - - - *d) Have you had sex with people at risk for HIV?* |  |  |  |  |
|  | Yes | No | | |
| - - *d1) Men who have sex with men* |  |  | | |
| - - *d2) People in prison* |  |  | | |
| - - - - *d3) People with sexually transmitted infections* |  |  | | |
| - - - - *d4) People injecting drugs* |  |  | | |
| - - - - *d5) Sex workers* |  |  | | |
| - - - - *d6) People from sub-Saharan Africa* |  |  | | |
| - - *How many sexual partners have you had during the last year?* | None (0) | | |  |
|  | One (1) | | |  |
|  | 2-5 (2) | | |  |
|  | >5 (3) | | |  |
|  | Number:……… | | |  |
|  | Don’t want to answer (5) | | |  |
| - 1. *Have you ever had a blood transfusion?* | Yes (1) | | |  |
|  | No (0) | | |  |
|  | Don’t want to answer (2) | | |  |
| *4.6 Have you ever injected yourself with drugs or any other substance sharing needles with other people?* | Yes (1) | | |  |
|  | No (0) | | |  |
|  | Don’t want to answer (2) | | |  |

Now a few questions about HIV testing in the Emergency Department during this visit.

| - 1. *Do you want to be tested for HIV now?* | Yes (1)  No (0) |  |
| --- | --- | --- |
|  |  |  |
| - 1. *If no, what is the main reason you do not want to be tested? (one response)* | Recent test (1) |  |
|  | I don’t think I am at risk (2) |  |
|  | I fear a positive result (3) |  |
|  | I prefer to focus on the reason I came in the ED (4) |  |
|  | Fear of needles (5) |  |
|  | Don’t want to answer (6) |  |
| - 1. *Are there other reasons why you don’t want the test? (Multiple responses allowed)* | Recent test (1) |  |
|  | I don’t think I am at risk (2) |  |
|  | I fear a positive result (3) |  |
|  | I prefer to focus on the reason I came in the ED (4) |  |
|  | Other (5)  ………………………………….. |  |
|  | Don’t want to answer (6) |  |
| - 1. *If yes, what is the main reason you want to be tested? (One response)* | I think I am at risk (1) |  |
|  | Following your questions, I think I am at risk (2) |  |
|  | I want to make sure I am HIV negative (3) |  |
|  | For fun (4) |  |
|  | This is an opportunity to do this once (5) |  |
|  | I consider the others, my partners, at risk (6) |  |
|  | need proof of a negative test for my gynecologist (7) |  |
|  | Don’t want to answer (8) |  |
| - 1. *Are there other reasons why you want to be tested? (Multiple responses allowed)* | I think I am at risk (1) |  |
|  | Following your questions, I think I am at risk (2) |  |
|  | I want to make sure I am HIV negative (3) |  |
|  | Other (4)  ………………………………….. |  |
|  | Don’t want to answer (5) |  |

Thank you for your valuable participation


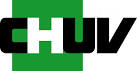

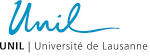


**Doctor questionnaire**

| Label | Acceptation of rapid HIV testing in the emergency department | Number: | |
| --- | --- | --- | --- |
|  | Master: | Date:  _ _ /_ _ /_ _ | Time:  _ _ : _ _ |

**Doctor data**

| 1.1. Nationality: | Switzerland (0) | |  | | South / Central America (3) |  |
| --- | --- | --- | --- | --- | --- | --- |
|  | Europe (1) | |  | | North America (4) |  |
|  | Africa (2) | |  | | Asia (5) |  |
|  | Country: | | | | | |
| 1.2. Sex: | Male (0) | |  | | Female (1) |  |
| 1.3. Title: | Junior doctor (0) | |  | | Service chief (2) |  |
|  | Senior doctor (1) | |  | | Other (3) |  |
| 1.4. Number of years of practice since graduation: |  | |  | |  |  |
| 1.5. Number of years of practice in the ED: |  | |  | |  |  |
| 1.6 Specialist service | Internal medicine |  | |  |  |  |
|  | Medical outpatients |  | |  |  |  |
|  | Orthopaedics |  | |  |  |  |
|  | General surgery surgery |  | |  |  |  |
|  | Emergency department |  | |  |  |  |

Thank you for agreeing to participate in this study.

We are interested in the HIV testing approaches in the emergency department. The following questions are about the consultation between you and the patient above (patient label).

| - 1. *Did you mention HIV with your patient?* | Yes (1) |  |
| --- | --- | --- |
|  | No (0) |  |
| - 1. *If not, for what reasons? (Multiple responses allowed)* | Recent test (1) |  |
|  | I don’t think that the patient is at risk (2) |  |
|  | I prefer to focus on the reason the patient came to the ED (3) |  |
|  | I don’t think it is relevant for the reason the patient came to the ED (4) |  |
|  | I have more urgent care to provide (5) |  |
|  | Conducting the test takes too long (6) |  |
|  | I will get the result after the patient leaves (7) |  |
|  | The test is expensive (8) |  |
|  | An HIV test is not recommended by the FOPH (9) |  |
|  | I didn’t think to bring up the subject (10) |  |
|  | I’m picking up this patient from another shift so I haven’t re-taken a full history (11) |  |
| - 1. *If yes, for what reasons? (Multiple responses allowed)* | An HIV test is recommended by the FOPH (1) |  |
|  | The patient wanted to be tested (2) |  |
|  | Other (3)  ……………………………………………………………. |  |
| - 1. *If you discussed HIV, did you offer a test?* | Yes (1) |  |
|  | No (0) |  |
| - 1. *If you didn’t offer a test, what were the reason(s) for not offering a test? (Multiple responses allowed)* | Recent test (1) |  |
|  | I prefer to focus on the reason the patient came to the ED (2) |  |
|  | I have more urgent care to provide (3) |  |
|  | Conducting the test takes too long (4) |  |
|  | I will get the result after the patient leaves (5) |  |
|  | The test is expensive (6) |  |
|  | Other (7)  ……………………………………………………………. |  |
| - 1. *Is an HIV test recommended by the 2013 FOPH recommendations?* | Yes (0) |  |
|  | No (1) |  |
|  | I don’t know (2) |  |
| - 1. *If yes, which one?* | AIDS-defining disease (1) |  |
|  | Disease indicating an immune system disorder (2) |  |
|  | Symptoms of primary HIV infection (3) |  |
|  | Disease that needs a treatment which may cause an immune system disorder (4) |  |
|  | Risk behaviour (5) |  |
|  | Population at risk (6) |  |
|  | Diseases, where the prevalence of undiagnosed HIV infections is probably> 0.5% (7) |  |
|  | Other (8) |  |
| *2.8 If yes, why didn’t you offer an HIV test? (Multiple responses allowed)* | I don’t feel comfortable (1) |  |
|  | I don’t have time (2) |  |
|  | I proposed that the test should be done elsewhere (3) |  |
|  | It is not the role of the ED to screen for HIV (4) |  |
|  | I lack training in HIV testing (5) |  |
|  | Patient has had recent test(6) |  |
|  | I prefer to focus on the reason that brought my patient to the ED (7) |  |
|  | I don’t think it relevant to the presenting complaint (8) |  |
|  | I have more urgent care to do (9) |  |
|  | I don’t consider the patient to be at risk (10) |  |
|  | Conducting the test takes too long (11) |  |
|  | I will get the result after the patient leaves (12) |  |
|  | The test is expensive (13) |  |
|  | I forgot to offer the test (14) |  |

Thank you for your valuable participation.

1. Resuscitation room [↑](#footnote-ref-2)
2. Opiates or other psychoactive drugs [↑](#footnote-ref-3)
