# Supplementary material for: Patient and doctor perspectives on HIV screening in the emergency department: A prospective cross-sectional study
Source: PLoS One. 2017 Jul 21;12(7):e0180389. doi: 10.1371/journal.pone.0180389 (PMC5521743; doi:10.1371/journal.pone.0180389)
Supplement: S2 Text — (DOC) [file pone.0180389.s002.doc]

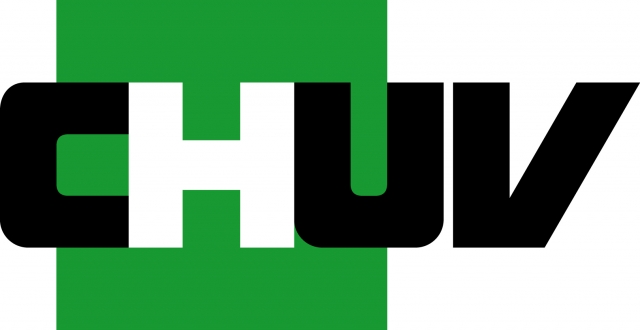
**HIV screening: who and when?**

2013 FOPH recommendations

| Table 1:  Potential indicator diseases of AIDS (the most common): **expressly recommend** testing |
| --- |

| **Tumours:** | - Cervical cancer - Non-Hodgkin lymphoma - Kaposi’s sarcoma |
| --- | --- |
| **Bacterial infections:** | - *Mycobacterium tuberculosis*, pulmonary or extra-pulmonary - *Mycobacterium avium* complex (MAC) or *Mycobacterium kansasii*, disseminated or extra-pulmonary - *Mycobacterium*, other species or unidentified species, disseminated or extra-pulmonary - Recurrent pneumonia (≥2 episodes within a 12-month period) - *Salmonella,* recurrent septicaemia in individuals <60 years |
| **Viral infections:** | - *Cytomegalovirus* (CMV), retinitis - CMV, other (except liver, spleen and lymph node) - *Herpes simplex*, ulcer(s) > 1 month / bronchitis / pneumonia - Progressive multifocal leucoencephalopathy |
| **Parasitic infections:** | - Cerebral *toxoplasmosis* - *Pneumocystis jirovecii* pneumonia - *Cryptosporidiosis-*associated diarrhoea, >1 month - *Isosporidiosis*, >1 month - *Leishmaniasis*, disseminated |
| **Fungal infections:** | - *Candidiasis,* oesophageal - C*andidiasis,* bronchial/tracheal/pulmonary - *Cryptococcosis,* extra-pulmonary |

| Table 2a:  Symptoms of primary HIV infection and indicator diseases for which the prevalence of undiagnosed HIV infection is >1%: **expressly** and ***immediately*** **recommend** testing in the presence of at least two of the following symptoms: |
| --- |

| - fever |
| --- |
| - (suspicion of) aseptic meningitis |
| - pathology suggesting mononucleosis |
| - disseminated lymphadenopathy |
| - pharyngitis with erythema |
| - skin rash |
| - reactive lymphocytes |

| Table 2b:  Other diseases for which the prevalence of undiagnosed HIV infection is probably >0.5%: **recommend** testing |
| --- |

| - Sexually transmitted infection | - Severe or atypical psoriasis |
| --- | --- |
| - Malignant lymphoma | - Guillain-Barré syndrome |
| - Anal carcinoma/dysplasia | - Mononeuropathy |
| - Cervical dysplasia | - Subcortical dementia |
| - Shingles in individuals <50 years | - Multiple sclerosis-like disease |
| - Hepatitis B or C (acute or chronic) | - Peripheral neuropathy |
| - Leucocytopaenia / unexplained thrombocytopaenia, >4 weeks | - Unexplained weight loss |
| - Seborrhoeic dermatitis / rash | - Unexplained lymphadenopathy |
| - Invasive pneumococcal disease | - Unexplained oral candidiasis |
| - Candidaemia | - Unexplained chronic diarrhoea |
| - Visceral leishmaniasis | - Unexplained chronic renal impairment |
| - Bronchial carcinoma | - Recurrent pneumonia (≥2 episodes within a 24-month period) |
| - Oral hairy leucoplakia |  |

| Table 3 :  Diseases for which an undiagnosed HIV infection can have particularly serious effects on the patient’s clinical management: **propose** testing |
| --- |

| - Conditions requiring aggressive immunosuppressive therapy: - Cancer - Transplant - Autoimmune disease |
| --- |
| - Invasive primary brain lesion |
| - Idiopathic thrombotic thrombocytopaenic purpura |

| Table 4:  Indications for **counselling** and **screening** proposed by the doctor: |
| --- |

| a) the patient belongs to a group with a high HIV prevalence: men who have sex with men, people who inject drugs, people from countries with high HIV prevalence (Sub-Saharan Africa); |
| --- |
| b) the patient evokes a history of high-risk sexual behaviour with a person known to be HIV positive or in a high HIV prevalence environment; |
| c) a sexually active person requests a screening test; |
| d) a person returning from travel requests a screening test. |
